# Supplementary material for: Differential requirements for cyclase-associated protein (CAP) in actin-dependent processes of Toxoplasma gondii
Source: eLife. 2019 Oct 2;8:e50598. doi: 10.7554/eLife.50598 (PMC6785269; doi:10.7554/eLife.50598)
Supplement: Supplementary file 4. [file elife-50598-supp4.docx]

Supplementary File 4. Key resources table

| **Key Resources Table** | | | | |
| --- | --- | --- | --- | --- |
| **Reagent type (species) or resource** | **Designation** | **Source or reference** | **Identifiers** | **Additional information** |
| Gene  (*Toxoplasma gondii*) | Cyclase-associated protein (CAP) | Lorestani et al., 2012 | TGGT1_310030 ([http://toxodb.org](http://toxodb.org/)) |  |
| Cell line  (*Homo* *sapiens*) | Human foreskin fibroblasts (HFF) | ATCC | ATCC Cat# SCRC-1041, RRID:CVCL_3285 | The cell line is available from the American Type Culture Collection (ATCC) |
| Cell line  (*Toxoplasma gondii*) | RH ∆*ku80* ∆*hxgprt* | Huynh & Carruthers, 2009 |  |  |
| Cell line  (*Toxoplasma gondii*) | RH DiCre ∆*ku80* ∆*hxgprt* | Andenmatten et al., 2013 |  | The first-generation DiCre-expressing cell line in *Toxoplasma gondii* |
| Cell line  (*Toxoplasma gondii*) | RH DiCre_T2A ∆*ku80* ∆*hxgprt* | This paper |  | The second-generation DiCre-expressing cell line in *Toxoplasma gondii* |
| Cell line  (*Toxoplasma gondii*) | LoxPCAP ; RH DiCre ∆*ku80* ∆*hxgprt_LoxCAP-HA* | This paper |  | The endogenous *CAP* gene was replaced with a floxed and HA-tagged *CAP* gene. |
| Cell line  (*Toxoplasma gondii*) | ∆CAP | This paper |  | A modified version of the “LoxPCAP” cell line:  The DiCre_T2A construct was integrated into the *Ku80* locus to allow for DiCre-mediated conditional excision.  The *CAP* gene was excised and an mCherry-expressing construct was integrated into the *Ku80* locus, replacing the DiCre_T2A construct. |
| Cell line  (*Toxoplasma gondii*) | ∆CAP^CAP^ | This paper |  | As described for the ∆CAP line, however a *CAP*-expressing construct (pUPRT_CAP) was integrated into the *UPRT* locus prior to *CAP* excision. |
| Cell line  (*Toxoplasma gondii*) | ∆CAP^shortCAP^ | This paper |  | As described for the ∆CAP line, however a short*CAP*-expressing construct (pUPRT_CAP_M1L) was integrated into the *UPRT* locus prior to *CAP* excision. |
| Cell line  (*Toxoplasma gondii*) | ∆CAP^longCAP^ | This paper |  | As described for the ∆CAP line, however a long*CAP*-expressing construct (pUPRT_CAP_M37L) was integrated into the *UPRT* locus prior to *CAP* excision. |
| Cell line  (*Toxoplasma gondii*) | Pru ∆*ku80* ∆*hxgprt* | Fox et al., 2011 |  |  |
| Cell line  (*Toxoplasma gondii*) | Pru ∆CAP | This paper |  | A modified version of the “Pru ∆*ku80* ∆*hxgprt*“ cell line:  The CAP gene was knocked out by replacement with a *HXGPRT* resistance cassette |
| Cell line  (*Toxoplasma gondii*) | Pru ∆CAP^CAP^ | This paper |  | A modified version of the “Pru ∆CAP” cell line:  A *CAP*-expressing construct (pUPRT_CAP) was integrated into the *UPRT* locus. |
| Cell line (*Toxoplasma gondii*) | Pru ∆CAP^shortCAP^ | This paper |  | A modified version of the “Pru ∆CAP” cell line:  A short*CAP*-expressing construct (pUPRT_CAP_M1L) was integrated into the *UPRT* locus. |
| Cell line (*Toxoplasma gondii*) | Pru ∆CAP^longCAP^ | This paper |  | A modified version of the “Pru ∆CAP” cell line:  A long*CAP*-expressing construct (pUPRT_CAP_M37L) was integrated into the *UPRT* locus. |
| Cell line (*Toxoplasma gondii*) | RH ∆*ku80* ∆*gra2* | Rommereim et al., 2016 |  |  |
| Cell line (*Toxoplasma gondii*) | ∆Gra2 | This paper |  | A modified version of the “RH ∆*ku80* ∆*gra2*” cell line:  An mCherry-expressing construct was integrated into the *UPRT* locus. |
| Cell line (*Toxoplasma gondii*) | CAP-HA ; CAP C-terminal endogenous HA-tagged line | This paper |  | A modified version of the “RH ∆*ku80* ∆*hxgprt*” cell line:  A C-terminal HA-tag was introduced to the *CAP* gene. |
| Cell line (*Toxoplasma gondii*) | RH Cb-Emerald ∆*ku80* | Periz et al., 2017 |  |  |
| Cell line (*Toxoplasma gondii*) | Cb-EmFP ∆CAP | This paper |  | A modified version of the “RH Cb-Emerald ∆*ku80*” cell line:  The CAP gene was knocked out by replacement with am mCherry-expressing cassette |
| Cell line (*Toxoplasma gondii*) | Cb-EmFP ∆CAP^CAP^ | This paper |  | A modified version of the “Cb-EmFP ∆CAP” cell line:  A *CAP*-expressing construct (pUPRT_CAP) was integrated into the *UPRT* locus. |
| Cell line (*Toxoplasma gondii*) | Cb-EmFP ∆CAP^shortCAP^ | This paper |  | A modified version of the “Cb-EmFP ∆CAP” cell line:  A short*CAP*-expressing construct (pUPRT_CAP_M1L) was integrated into the *UPRT* locus. |
| Cell line (*Toxoplasma gondii*) | Cb-EmFP ∆CAP^longCAP^ | This paper |  | A modified version of the “Cb-EmFP ∆CAP” cell line:  A long*CAP*-expressing construct (pUPRT_CAP_M37L) was integrated into the *UPRT* locus. |
| Antibody | Rat anti- haemagglutinin (HA) (monoclonal) | Roche | Roche Cat#  11867423001 | Monoclonal (clone 3F10)  IFA (1:1000), WB: 1:1000) |
| Antibody | Mouse anti-*Toxoplasma* (38 kDa protein)  (monoclonal) | Abcam | #ab130884  (discontinued) | Monoclonal (clone B1247M)  IFA (1:1000 -1:20000) |
| Antibody | Mouse anti-*Toxoplasma*  (monoclonal) | Abcam | #ab8313 | Monoclonal (clone TP3)  This antibody reacts with p30 membrane protein  IFA (1:1000), WB: 1:1000) |
| Antibody | Rabbit anti-TgRON4  (monoclonal) | Leriche & Dubremetz, 1991 |  | Monoclonal (clone T5 4H1)  IFA (1:2000) |
| Antibody | Rabbit anti-TgCAP  (polyclonal) | This paper |  | Polyclonal  See Materials & Methods section: “Generation of *T.gondii* CAP antibody”  IFA (1:2000), WB: 1:2000) |
| Chemical compound | 5-Benzyl-3-isopropyl-1H-pyrazolo[4,3-d]pyrimidin-7(6H)-one (BIPPO) | Howard et al., 2015 |  | Used at a final concentration of 50 µM.  The reagent used here was synthesised in-house by the Peptide Chemistry science technology platform, The Francis Crick Institute. |
| Software, algorithm | Prism 8 (version 8.1.1) | GraphPad Software, Inc. |  | Commercial software for statistical analysis |
